# The role of brain mechanisms in diabetic peripheral neuropathy: recent advances and comprehensive analysis

**DOI:** 10.3389/fncel.2025.1637357

**Published:** 2025-10-29

**Authors:** Min Wei, Ye Jiang, Jiayin Shou, Guogang Xing, Min Li

**Affiliations:** 1 Department of Anesthesiology, Peking University Third Hospital, Beijing, China; 2 Neuroscience Research Institute, Peking University, Beijing, China; 3 Department of Neurobiology, School of Basic Medical Sciences, Peking University Health Science Center, Beijing, China; 4 Key Laboratory for Neuroscience, Ministry of Education of China and National Health Commission of China, Beijing, China

**Keywords:** diabetic peripheral neuropathy, brain mechanisms, neuroimaging, neuroinflammation, neuromodulation

## Abstract

Diabetic peripheral neuropathy (DPN), a prevalent and debilitating complication of diabetes, involves complex interactions between peripheral nerve damage and central nervous system (CNS) dysfunction. While traditional research has focused on peripheral and spinal mechanisms, emerging evidence highlights that the brain plays a critical role in the development of painful DPN. This review synthesizes recent advances from neuroimaging, spectroscopy, and preclinical studies to delineate structural, functional, and neurochemical alterations in the central nervous system associated with DPN. Patients exhibit cortical thinning, subcortical atrophy, and disrupted connectivity in sensory, affective, and cognitive networks, accompanied by metabolic imbalances and excitatory–inhibitory neurotransmitter shifts. Preclinical models further implicate maladaptive plasticity, microglial activation, and region-specific astrocytic responses in amplifying central sensitization and pain chronicity. These mechanistic insights underscore the central nervous system as a therapeutic target. Non-invasive neuromodulation techniques, such as repetitive transcranial magnetic stimulation, and brain-directed pharmacological strategies show promising but preliminary benefits in alleviating neuropathic pain. Understanding the interplay between peripheral injury and brain dysfunction in DPN not only broadens the conceptual framework of its pathophysiology but also provides a foundation for developing novel interventions aimed at restoring central network balance and improving patient outcomes.

## Graphical abstract

ACC, anterior cingulate cortex; PCC, posterior cingulate cortex; PFC, prefrontal cortex; M1, primary motor cortex; S1, primary somatosensory cortex; PVT, paraventricular thalamus; vlPAG, ventrolateral periaqueductal gray; LC, locus coeruleus; RVM, rostral ventromedial medulla; A5, A5 noradrenergic cell group; Glu, glutamate; PKMζ, protein kinase M zeta; NA, noradrenaline; 5-HT, 5-hydroxytryptamine (serotonin); CXCL12, C-X-C motif chemokine ligand 12; CXCR4, C-X-C motif chemokine receptor 4; ROS, reactive oxygen species; TSPO, 18-kDa translocator protein; Iba-1, ionized calcium-binding adaptor molecule 1; GFAP, glial fibrillary acidic protein; TNF-α, tumor necrosis factor alpha; IL-1β, interleukin-1 beta.

## 1 Introduction

Diabetes mellitus is a chronic and complex metabolic disorder characterized by persistent hyperglycemia resulting from pancreatic *β*-cell dysfunction and insulin resistance. Over time, this condition leads to absolute or relative insulin deficiency, which contributes to a wide range of systemic complications. With rising global prevalence, diabetes has become one of the most pressing public health challenges. As of 2021, there were 529 million individuals diagnosed with diabetes, and this figure is projected to escalate to 1.3 billion by 2050, driven by an aging population, sedentary lifestyles, and dietary changes (Cho et al., 2018; Cole and Florez, 2020; Walker et al., 2023). The global economic and healthcare burden of diabetes is immense, requiring sustained efforts to develop effective prevention and management strategies (NCD Risk Factor Collaboration, 2016).

One of the most debilitating complications of diabetes is diabetic peripheral neuropathy (DPN), a progressive microvascular complication affecting approximately 50% of diabetic patients (Faselis et al., 2020). Among these patients, 15–25% experience painful DPN (Shillo et al., 2019), which is characterized by chronic and persistent pain that exacerbates the emotional and psychological challenges of managing diabetes (Dyck et al., 1993; Pouwer et al., 2024). Despite the profound clinical impact of DPN, effective treatment options remain limited, primarily due to an incomplete understanding of its pathogenesis.

Traditional research on DPN has predominantly focused on the peripheral nervous system and spinal pathways (Yang et al., 2025). These studies have identified key mechanisms such as peripheral nerve damage, microvascular complications, and oxidative stress. However, emerging evidence highlights the involvement of the brain in the integration and modulation of pain signals in DPN (Kapur, 2003). Pain perception is not solely determined at the peripheral or spinal level, but is shaped by a distributed brain network encompassing the cortex, thalamus, hippocampus, and brainstem nuclei (Yang et al., 2019; Latremoliere and Woolf, 2009). This constellation of brain regions, often referred to as the “pain matrix,” integrates sensory-discriminative, affective, and cognitive dimensions of pain processing (Garcia-Larrea and Peyron, 2013; Greig et al., 2014). Under diabetic conditions, structural and functional alterations in these brain areas can amplify pain sensitivity, disrupt descending inhibitory pathways, and contribute to the persistence of neuropathic pain (Segerdahl et al., 2018). While peripheral mechanisms initiate aberrant nociceptive signaling, central changes amplify and modulate these inputs, highlighting the complementary yet distinct contributions of peripheral and central processes to DPN pathophysiology (Schaible, 2007; Sloan et al., 2021).

Recognizing DPN as a condition that involves peripheral, spinal, and brain changes is essential for advancing our understanding and treatment of this complex disease. This review aims to provide a comprehensive overview of brain mechanisms implicated in painful DPN. Clarifying these brain-specific contributions may facilitate the development of novel neuromodulator or pharmacological interventions to better manage neuropathic pain in diabetic patients.

## 2 Structural changes in the brain induced by DPN

DPN is increasingly recognized as a condition involving not only peripheral nerve damage but also central nervous system (CNS) alterations (Tesfaye et al., 2016; Zang et al., 2023). Accumulating neuroimaging evidence indicates that DPN is associated with significant structural changes in the brain, including cortical thinning, gray matter atrophy, and regional volume loss (Selvarajah et al., 2023; Zhang et al., 2020; Selvarajah et al., 2014). These alterations are closely linked to the sensory and affective manifestations of neuropathic pain (Zang et al., 2023), highlighting the importance of brain involvement in the pathogenesis and clinical expression of DPN (Yang et al., 2025).

High-resolution structural magnetic resonance imaging (MRI), particularly surface-based morphometry (SBM) and voxel-based morphometry (VBM) have enabled accurate evaluation of cortical morphology in DPN patients (Zhang et al., 2020; Tae et al., 2025; Scheliga et al., 2024). Cortical thickness, typically assessed by SBM, measures the distance between the white matter and pial surfaces, whereas gray matter volume, quantified through VBM, incorporates both thickness and surface area (Clarkson et al., 2011; Tang et al., 2018). Although related, these markers are derived through distinct computational approaches and may reveal complementary, non-redundant aspects of cortical pathology (Clarkson et al., 2011; Schwarz et al., 2016).

### 2.1 Cortical alterations: thinning and volume loss

Cortical alterations in DPN primarily manifest as reductions in cortical thickness and volume, reflecting neuronal loss, dendritic retraction, or glial changes (Muhlau et al., 2007; Vidal-Pineiro et al., 2020). These structural deficits are commonly observed in brain regions involved in pain processing, sensorimotor integration, emotional regulation, and attentional modulation, and are more pronounced in painful DPN, suggesting a central contribution to pain chronification (Selvarajah et al., 2023; Davis and Moayedi, 2013; Tracey and Mantyh, 2007; Hostrup et al., 2025).

Cortical thinning in DPN reflects region-specific reductions in thickness, often seen as localized microstructural damage. Notably, cortical thinning has been reported in several key brain regions, including the primary somatosensory cortex (S1, postcentral gyrus) (Selvarajah et al., 2023; Zhang et al., 2020; Selvarajah et al., 2014; Hostrup et al., 2025; Selvarajah et al., 2019; Hansen et al., 2022a; Frokjaer et al., 2013), primary motor cortex (M1, precentral gyrus) (Selvarajah et al., 2023; Zhang et al., 2020; Hansen et al., 2022a), insular cortex (Selvarajah et al., 2023; Zhang et al., 2020), anterior cingulate cortex (ACC) (Selvarajah et al., 2023; Zhang et al., 2020), middle cingulate cortex (Zhang et al., 2020), superior parietal gyrus/lobule (Hansen et al., 2022b), and supramarginal gyrus (Selvarajah et al., 2014; Hostrup et al., 2025). These changes are typically more marked in painful DPN and are thought to reflect maladaptive plasticity triggered by chronic peripheral nerve injury (Li et al., 2016). Thinning in regions such as the insula and S1 has been associated with enhanced pain intensity and disrupted sensory-emotional integration, potentially contributing to central sensitization (Selvarajah et al., 2023; Zhang et al., 2020; Hansen et al., 2022b; He et al., 2025; Chao et al., 2022a). Cortical thinning is commonly associated with normal aging (Cao et al., 2017) and is often accelerated in neurodegenerative diseases such as Alzheimer’s disease (Wu et al., 2021). In the context of DPN, however, cortical thinning likely reflects a combination of diabetes-related systemic effects and central nervous system adaptations to chronic neuropathic pain (Hostrup et al., 2025).

Cortical volume loss, on the other hand, integrates cortical thickness with surface area and folding patterns, offering a broader perspective on atrophy (Winkler et al., 2010; Lemaitre et al., 2012). Reductions in gray matter volume have been identified in the S1 (Selvarajah et al., 2014; Hansen et al., 2022a), M1 (Hansen et al., 2022a), cingulate cortex (Selvarajah et al., 2023; Selvarajah et al., 2014), supramarginal gyrus (Selvarajah et al., 2014), and inferior/superior occipital gyrus (Hansen et al., 2022a). These volumetric losses often overlap with thinning regions but may also indicate more extensive neuronal compromise. In painful DPN, greater volume loss in areas such as the ACC has been linked to intensified affective symptoms like distress and catastrophizing (Penzo and Gao, 2021; Sifuentes-Franco et al., 2017), while atrophy in the posterior cingulate cortex and parietal regions may impair the sensory-discriminative components of pain perception. Mechanistically, chronic hyperglycemia, oxidative stress (Penzo and Gao, 2021; Sifuentes-Franco et al., 2017), and microvascular injury (Van Dam et al., 2013) may drive these structural alterations through neurodegenerative and neuroinflammatory pathways (see Table 1).

**Table 1:** Summary of structural, connectivity, and mechanistic changes in the brain in DPN.

Change type	Region/structure	Functional/pathological significance	Mechanism/key findings	References
Structural changes—cortex	Sensory and motor cortices (S1, M1, superior parietal/supramarginal gyrus)	Thinning/volume loss; disrupts sensory-motor integration and increases pain intensity	Maladaptive plasticity from hyperglycemia/oxidative stress; impairs sensory processing	Selvarajah et al. (2023), Zhang et al. (2020), Selvarajah et al. (2014), Hostrup et al. (2025), Selvarajah et al. (2019), Hansen et al. (2022a), Frokjaer et al. (2013), Hansen et al. (2022b)
	Affective and Integrative Cortices (ACC/Midcingulate, Insula)	Thinning/volume loss; abnormal affective regulation; heightens distress/catastrophizing	Neuronal degeneration and central sensitization; disrupts emotional integration	Selvarajah et al. (2023), Zhang et al. (2020), Selvarajah et al. (2014)
Structural changes—subcortical	Thalamus and Basal Ganglia (Putamen/Caudate)	Volume loss (more in painless DPN); impairs sensory/motor modulation and emotion-related pain	Thalamo-cortical dysfunction and neurodegeneration; phenotype-specific amplification	Selvarajah et al. (2023), Zhang et al. (2020), Selvarajah et al. (2014), Selvarajah et al. (2019), Hansen et al. (2022b), He et al. (2025)
	Limbic Structures (Hippocampus/Amygdala)	Volume loss; impairs emotional regulation and inhibition	Neuroinflammation/degeneration; promotes pain persistence and anxiety	Zhang et al. (2020)
Connectivity Changes	Thalamocortical and Thalamus–S1 Networks	FC ↓; impairs sensory transmission and amplifies pain	Thalamocortical dysrhythmia; linked to peripheral damage	Cauda et al. (2009), Teh et al. (2021)
	Thalamus–Insula and Thalamus–Parietal/Occipital	FC ↑; heightens affective-attentional and aberrant sensory processing	Hyperactivation in pain circuits; correlates with pain scores	Teh et al. (2021), Chao et al. (2022b), Croosu et al. (2023), Liu et al. (2021)
	Limbic-ACC/Hippocampus/Temporal Lobe	Connectivity ↓, efficiency ↓; impairs emotional control	White matter damage; reduces network integration	Chao et al. (2022a)
Mechanistic findings (Rodents)	Synaptic/Neurotransmitter Dysregulation (ACC, LC, PAG)	Glutamate/PKMζ ↑, output ↓, imbalance; causes sensitization and inhibition loss	Synaptic potentiation; IGF-1 restores function/reduces hyperalgesia	Li et al. (2014), Li et al. (2022), Suehiro et al. (2013), Mesa-Lombardo et al. (2023), Morgado et al. (2011)
	Microglial Activation (ACC, Cortex, Thalamus, RVM)	Activation ↑, ON-cell ↑; enhances signaling and facilitation	Neuroinflammation via CXCL12/CXCR4 and TRPV1/5-HT3; amplifies nociception	Wang et al. (2024), Zhang et al. (2022), Song et al. (2023), Silva et al. (2016)
	Astrocytic Activation (Motor cortex, PVT, vlPAG, Hippocampus)	GFAP/P2X7/Ca^2+^ ↑; amplifies pain and inflammation	Cytokine release (TNF-*α*/IL-1β); inhibition alleviates allodynia; dihydromyricetin protects	Lu et al. (2021), Chen et al. (2025), Yang L. et al. (2022), Ge et al. (2020)

ACC, anterior cingulate cortex; CXCL12, chemokine ligand 12; CXCR4, C-X-C chemokine receptor type 4; DPN, diabetic peripheral neuropathy; FC, functional connectivity; GFAP, glial fibrillary acidic protein; IGF-1, insulin-like growth factor 1; IL-1β, interleukin-1 beta; LC, locus coeruleus; M1, primary motor cortex; P2X7, purinergic receptor P2X7; PAG, periaqueductal gray; PKMζ, protein kinase M zeta; PVT, paraventricular thalamic nucleus; RVM, rostral ventromedial medulla; S1, primary somatosensory cortex; TNF-α, tumor necrosis factor alpha; TRPV1, transient receptor potential vanilloid 1; vlPAG, ventrolateral periaqueductal gray; 5-HT3, 5-hydroxytryptamine receptor 3.

### 2.2 Subcortical alterations: volume loss

Subcortical volume loss, often quantified through voxel-based morphometry, has been consistently reported in DPN, particularly affecting deep gray matter nuclei integral to pain modulation and sensory processing (Selvarajah et al., 2014; Hansen et al., 2022b). Significant atrophy has been identified in the thalamus (Selvarajah et al., 2023; Zhang et al., 2020; Selvarajah et al., 2014; Selvarajah et al., 2019; Hansen et al., 2022b), putamen (Zhang et al., 2020; Hansen et al., 2022b; He et al., 2025), caudate nucleus (Zhang et al., 2020; Hansen et al., 2022b; He et al., 2025), pallidum (Zhang et al., 2020; He et al., 2025), hippocampus (Zhang et al., 2020), and amygdala (Zhang et al., 2020)—critical nodes in ascending and descending pain pathways that facilitate sensory processing, motor-sensory integration, autonomic regulation, and pain inhibition. Reductions in putamen and caudate nucleus volumes may disrupt basal ganglia-mediated modulation of sensorimotor and affective pain components (Chudler and Dong, 1995), potentially exacerbating movement-related symptoms and emotional distress in DPN (Zhang et al., 2020; Hansen et al., 2022b; He et al., 2025). Similarly, atrophy in the hippocampus and amygdala could impair descending inhibitory control, promoting pain persistence and contributing to associated emotional dysregulation, such as anxiety (Zhang et al., 2020). Phenotype-specific patterns have also emerged. For instance, thalamic volume appears more reduced in painless DPN than in painful DPN, particularly on the right side (Novo et al., 2022). Conversely, painful DPN may involve relatively preserved thalamic structure but dysfunctional thalamo-cortical signaling, contributing to abnormal nociceptive amplification (Hansen et al., 2022a; Novo et al., 2022).

## 3 Brain dysfunction induced by DPN

Advances in neuroimaging techniques, such as functional magnetic resonance imaging (fMRI), have facilitated the identification of microstructural and functional impairments within the central nervous system (Yen et al., 2023; Ugurbil et al., 2003). Using fMRI, functional disruptions in brain networks involved in the affective and cognitive modulation of pain can be revealed (Zhang L. B. et al., 2024; Martucci and Mackey, 2018).

### 3.1 Resting-state functional connectivity: disruptions in pain and sensory networks

Resting-state fMRI is a powerful tool used to evaluate spontaneous brain activity by measuring functional connectivity (Barkhof et al., 2014). RS-fMRI detects low-frequency blood-oxygen-level-dependent (BOLD) fluctuations, allowing for analysis of temporal correlations between spatially distinct brain regions—termed functional connectivity (FC)—and thus provides insights into brain network alterations without task-related stimulation (Baracchini et al., 2021; Allen et al., 2014).

One of the earliest studies applying RS-fMRI in DPN demonstrated significantly reduced thalamocortical functional connectivity in patients with painful DPN (PDN) (Cauda et al., 2009). Specifically, FC between the ventral posterior lateral (VPL) and mediodorsal thalamic nuclei and the S1 was diminished, supporting the notion that chronic pain disrupts thalamocortical feedback loops, a concept known as thalamocortical dysrhythmia (Cauda et al., 2009). Concurrently, modulation of the dorsolateral prefrontal cortex–anterior cingulate cortex–medial thalamus loop has been proposed in PDN, consistent with decreased anterior cingulate perfusion during rest.

A more recent study stratified DPN patients by nociceptor phenotype and found a double dissociation in thalamocortical FC: thalamus–insula FC was positively associated with neuropathic pain scores, while thalamus–somatosensory cortex FC was inversely correlated with the severity of peripheral nerve damage (Teh et al., 2021). The insula, implicated in affective and attentional pain processing, may be hyperactive in pain-promoting circuits among individuals with preserved nociceptor input.

In a diffusion MRI study assessing structural connectivity (SC), reduced thalamic and hypothalamic SC with the amygdala and insula has been reported in PDN, compared to both painless DPN and healthy controls (Chao et al., 2022b). Lower SC in the anterior cingulate cortex correlated with greater autonomic dysfunction, linking limbic disconnection to both pain and dysautonomia.

Furthermore, FC alterations appear phenotype-specific. A 2023 study found that, compared to painful DPN and controls, type 1 diabetes patients without neuropathy exhibited hyperconnectivity between the thalamus/postcentral gyrus and motor areas (Croosu et al., 2023). In contrast, PDN was associated with reduced FC in these pathways, with stronger associations observed between thalamic FC and both pain scores and nerve conduction deficits.

Another RS-fMRI study reported enhanced thalamic FC with the parietal and occipital cortices in patients with type 2 diabetes and PDN, implicating thalamoparietal overactivation in the pathophysiology of pain (Liu et al., 2021).

Complementing functional studies, graph theory analysis of structural networks constructed from diffusion tractography showed PDN-specific reductions in white matter connectivity within the insula, hippocampus, and temporal lobe, along with decreased global efficiency and betweenness centrality—indicative of widespread disintegration of integrative brain networks (Chao et al., 2022a).

Collectively, these studies demonstrate that PDN is associated with altered thalamocortical and limbic connectivity, involving both sensory-discriminative and affective-emotional components of pain. Such connectivity patterns are further modulated by disease phenotype and severity, suggesting potential for FC-based biomarkers in diagnosis and monitoring of DPN.

### 3.2 Task-based imaging: cortical reorganization and pain modulation

Task-based fMRI approach measures brain activity in response to specific stimuli or tasks, providing insights into the functional reorganization of neural circuits in response to sensory inputs or motor demands (Huang et al., 2024).

Studies using this technique have revealed how the brain responds to sensory stimuli in diabetic peripheral neuropathy. During thermal nociceptive stimulation, patients with severe diabetic distal symmetrical polyneuropathy exhibit expanded activation of the primary somatosensory cortex, with abnormal representations extending into non-somatotopic areas such as the facial and lip cortices (Selvarajah et al., 2019). This pattern reflects central plasticity resulting from peripheral deafferentation and suggests a cortical spread of nociceptive encoding.

Additionally, in response to noxious heat stimulation, patients with painful DPN show increased BOLD activation in the ACC, anterior insula, and supplementary motor areas—changes that positively correlate with pain intensity and affective distress (Tseng et al., 2013). In contrast, patients with painless DPN display reduced activation in the ACC and S1, highlighting distinct patterns of central reorganization between DPN subtypes.

Further supporting this, a study (Li et al., 2018) found that compared to healthy individuals and diabetic patients without neuropathy, those with DPN showed significantly stronger activation in somatosensory-related regions—including the right insula, left caudate nucleus, frontal gyrus, and cingulate cortex—in response to thermal stimuli. These findings underscore the potential of task-based fMRI as a sensitive tool for detecting early central nervous system involvement in DPN.

### 3.3 Neurochemical and metabolic alterations in the brain: insights from magnetic resonance spectroscopy (MRS)

MRS studies have been used to identify metabolic abnormalities in key brain regions to understand changes across chronic pain conditions (Cruz-Almeida and Porges, 2021). In addition to functional imaging insights, clinical metabolic and neurochemical assessments further implicate central involvement in DPN (Zhao et al., 2018).

Sloan *et al.* demonstrated that patients with painful DPN exhibit significantly reduced phosphocreatine-to-ATP (PCr: ATP) ratios in the primary somatosensory (S1) cortex compared with painless DPN, indicating higher cortical energy consumption in pain phenotypes (Sloan et al., 2023). Moreover, lower PCr: ATP ratios correlated with greater pain intensity, suggesting that altered cortical bioenergetics may serve as a biomarker of painful DPN.

Beyond high-energy phosphate changes, several MRS studies have identified alterations in metabolites reflecting neuronal integrity, glial activity, and membrane turnover. Selvarajah et al. (2008) found preserved S1 cortical metabolites but reduced thalamic N-acetylaspartate (NAA) -to-creatine (Cr) ratio in advanced painless DPN, with preservation in painful DPN, suggesting that intact thalamic neuronal function may be a prerequisite for pain perception. Similarly, Hansen *et al.* reported decreased NAA/Cr ratios and increased myo-inositol/Cr in parietal and cingulate regions in type 1 diabetes, with greater reductions linked to more severe DPN (Hansen et al., 2024). Painful DPN was further associated with increased glycerophosphocholine/Cr and elevated thalamic glutamate, indicating enhanced membrane turnover and heightened excitatory neurotransmission in pain phenotypes.

Altered neurotransmitter balance has also been reported in DPN. Petrou *et al.* found significantly higher glutamate/glutamine and lower *γ*-aminobutyric acid (GABA) levels in the posterior insula of patients with diabetic neuropathy and positive sensory symptoms compared with healthy controls, indicating an excitatory/inhibitory imbalance in key pain-processing areas (Petrou et al., 2012). Supporting this, Shillo *et al.* reported that painless DPN was characterized by the lowest thalamic GABA: H_2_O ratio compared with both healthy volunteers and diabetes patients without DPN, whereas painful DPN maintained partially preserved GABA levels, suggesting that central GABAergic pathways may be critical for neuropathic pain mechanisms (Shillo et al., 2024).

Taken together, clinical neuroimaging studies consistently support phenotype-specific central alterations in DPN, suggesting that painful and painless subtypes may follow partially distinct neurobiological trajectories. This distinction provides an important framework for interpreting mechanistic findings from preclinical models.

## 4 Brain-centered mechanistic findings from rodent models of DPN

To complement clinical imaging findings, preclinical studies in rodent models have been widely used to explore brain-specific mechanisms underlying diabetic neuropathic pain. These studies help elucidate cellular and molecular processes that are difficult to access in human subjects. As illustrated in Graphical abstract, the following sections summarize key brain mechanisms identified in animal models of DPN.

### 4.1 Synaptic and neurotransmitter dysregulation

Preclinical studies provide mechanistic validation for clinical findings and offer deeper insight into specific neural circuits involved in DPN.

In diabetic rodent models, elevated glutamatergic activity has been observed in ACC neurons (Li et al., 2014). This is driven by both increased presynaptic glutamate release and enhanced postsynaptic receptor responsiveness, accompanied by elevated PKMζ phosphorylation. Pharmacological blockade of PKMζ reversed thermal hyperalgesia and mechanical allodynia and normalized synaptic activity, underscoring its role in central sensitization (Li et al., 2014).

Further studies highlight dysfunction in descending pain modulation systems, particularly the locus coeruleus (LC) -spinal noradrenergic circuits. In DPN rats, reduced LC output correlates with diminished inhibition of spinal nociceptive transmission, poor analgesic efficacy, and persistent spinal glial activation (Li et al., 2022; Suehiro et al., 2013; Mesa-Lombardo et al., 2023). Moreover, LC dysfunction impairs regulation of emotional tone, exacerbating depressive and anxiety-like behaviors, consistent with clinical affective symptoms in DPN (Alba-Delgado et al., 2016; Espana et al., 2024).

Similarly, the periaqueductal gray (PAG)—a central hub for pain modulation—exhibits neurotransmitter dysregulation in DPN models (Morgado et al., 2011). Serotonin and noradrenaline imbalances impair descending inhibition, while insulin-like growth factor 1 (IGF-1) treatment has been shown to restore neurotransmitter balance within the PAG, leading to significant reductions in mechanical hyperalgesia (Morgado et al., 2011).

### 4.2 Microglial activation: linking neuroinflammation to neuronal injury

Microglia, the primary immune effector cells of the CNS, are central to neuroinflammation, and play a critical role in the central mechanisms underlying DPN (Wang et al., 2024).

In one study, positron emission tomography/computed tomography (PET/CT) imaging revealed increased translocator protein expression in the cortex and thalamus of diabetic rats, coupled with higher numbers of Iba-1-positive microglial cells (Zhang et al., 2022). These alterations are correlated with reduced mechanical and thermal pain thresholds, underscoring the role of microglia in pain hypersensitivity.

Further evidence from streptozotocin (STZ)-induced diabetic mouse models reveals marked microglial activation in the ACC, along with upregulated expression of the chemokine CXCL12 and its neuronal receptor CXCR4 (Song et al., 2023). This CXCL12/CXCR4 signaling enhances glutamatergic neuron excitability in the ACC, contributing to central sensitization and persistent mechanical pain in DPN.

The rostral ventromedial medulla (RVM), a brainstem center involved in descending pain facilitation, also shows early-stage microglial reactivity in DPN (Silva et al., 2016). Diabetic rodents demonstrate increased ON-cell activity, spinal 5-HT3 receptor expression, and TRPV1 upregulation, all of which facilitate nociceptive signal amplification (Silva et al., 2016). As DPN progresses, oxidative stress and microglial activation within the RVM further exacerbate neuroinflammation and neurodegeneration.

### 4.3 Region-specific astrocytic responses in central pain processing

Astrocytes, the most abundant glial cells in the CNS, play a crucial role in maintaining homeostasis, regulating neuronal activity, and mediating inflammatory responses (Giovannoni and Quintana, 2020). In DPN, astrocytic changes exhibit regional heterogeneity, with their activation contributing differentially to central pain amplification across pain-modulating brain structures (Cheng et al., 2022).

In STZ-induced diabetic models, the significantly increased expression of glial fibrillary acidic protein (GFAP)—a hallmark of astrocyte activation—has been detected in the motor cortex following the onset of DPN, indicating the involvement of motor cortex astrocytes in the pathogenesis of DPN (Lu et al., 2021). Functional inhibition of astrocytes in this region alleviated mechanical allodynia, alongside reduced expression of pro-inflammatory cytokine, including TNF-*α* and IL-1β.

The paraventricular thalamic nucleus (PVT), a midline thalamic structure crucial for sensory and nociceptive signal processing (Penzo and Gao, 2021), also exhibits significant astrocytic activation during DPN. In a study of DPN male rat, astrocytic activity within the PVT is markedly upregulated, accompanied by decreased neuronal activity at around 14 days following STZ administration (Chen et al., 2025). Chemogenetic inhibition of astrocytes in this region alleviates mechanical allodynia, whereas artificial activation in healthy rodents is sufficient to induce pain behavior.

Astrocyte activation is also evident in the ventrolateral periaqueductal gray (vlPAG), a core component of the descending pain inhibitory pathway (Tracey and Mantyh, 2007). Astrocytes in this region exhibit time-dependent activation and morphological changes, becoming significantly reactive after 14 days of STZ administration (Yang L. et al., 2022). Chemogenetic activation of vlPAG astrocytes in naive rats induces pain-like behaviors and aversion, while their inhibition in DPN model rats alleviates mechanical hypersensitivity and promotes preference behavior.

*In vitro* studies mimicking DPN with depression have shown that high glucose, substance P, and corticosterone exposure lead to astrocyte damage (Ge et al., 2020). This is marked by upregulated P2X7 receptor expression, elevated TNF-α and IL-1β levels, increased cytoplasmic Ca^2+^, and enhanced ERK1/2 phosphorylation. Notably, dihydromyricetin treatment protects primary hippocampal astrocytes from cytotoxicity and reduces inflammation, underscoring the importance of targeting astrocyte dysfunction to manage comorbidities in DPN.

## 5 Potential therapeutic strategies

The neuroimaging and mechanistic findings summarized above not only deepen our understanding of central alterations in DPN but also provide a critical basis for therapeutic development. Cortical reorganization and disrupted network activity revealed by neuroimaging point to neuromodulation of specific brain regions as a potential strategy (Chao et al., 2022a; Zeng et al., 2020; Li and Gao, 2025), while evidence of neuroinflammation and glial activation highlights molecular targets for pharmacological intervention (Kaur et al., 2025; Cheng et al., 2024). Building on these insights, the following section discusses emerging therapeutic approaches that exemplify how mechanistic discoveries can be translated into clinical strategies.

### 5.1 Transcranial non-invasive treatment of DPN

The following section summarizes findings primarily derived from clinical studies in human participants, focusing on non-invasive neuromodulatory approaches, particularly those targeting central pain processing pathways (Knotkova et al., 2021).

Transcranial magnetic stimulation (TMS), especially in the form of repetitive protocols (rTMS), utilizes pulsed magnetic fields to generate localized electric currents in targeted cortical areas (Davidson et al., 2024). This technique allows for precise modulation of neural circuits involved in pain perception and emotional regulation (Weise et al., 2023; Jayathilake et al., 2025).

A single-blinded randomized controlled trial investigated prolonged continuous theta burst stimulation (pcTBS) targeting both M1 and dorsolateral PFC in neuropathic pain patients (Thakkar et al., 2023). Neurophysiological assessments revealed modulation of motor corticospinal excitability and GABAergic activity, while no significant changes were observed in ascending/descending endogenous pain modulation systems. Although standardized pain scores remained unchanged, self-reported acute pain intensity showed a 13% improvement post-intervention, suggesting transient analgesic effects.

Similarly, another study evaluated the effect of a single-session pcTBS targeting the same cortical regions in patients with DPN (Thakkar et al., 2024). Findings indicated multidimensional analgesic effects, with improvements reported across sensory-discriminative, affective-motivational, and cognitive-evaluative domains of pain perception. Importantly, no adverse events were observed within 24 h post-intervention, supporting the safety and clinical feasibility of this non-invasive approach.

Further evidence was provided by a study that assessed the therapeutic efficacy of rTMS in DPN patients immediately after treatment and at a one-week follow-up (Yang S. et al., 2022). The results showed a sustained reduction in pain intensity along with improvements in overall quality of life. Specifically, both physical and mental component scores showed significant enhancements, underscoring the potential of rTMS not only to alleviate pain but also to improve psychosocial well-being.

These preliminary findings support transcranial non-invasive neuromodulation as a promising adjunctive strategy for the treatment of DPN. However, further research is needed to determine optimal stimulation parameters, treatment frequency, and patient selection criteria, which will be critical for maximizing clinical outcomes and individualizing therapy.

### 5.2 Brain-targeting compounds

Emerging research highlights the therapeutic potential of diverse compounds—ranging from natural phytochemicals to synthetic drugs—for the treatment of DPN (Qureshi et al., 2022; Arora et al., 2021; Zhang E. X. et al., 2024). However, the studies discussed in this section are derived entirely from preclinical/basic research in animal models. While they offer important mechanistic insights, their direct applicability to clinical practice remains to be established through rigorous translational and clinical studies.

Given the well-established role of neuroinflammation in DPN pathogenesis, strategies aimed at modulating central glial activation, particularly astrocytes and microglia, have garnered increasing interest (Llorian-Salvador et al., 2024). One promising example is Koumine, a bioactive alkaloid derived from *Gelsemium elegans* Benth., which has demonstrated anti-inflammatory and analgesic effects in preclinical studies (Que et al., 2021). Its therapeutic actions include the suppression of astrocyte activation in the basolateral amygdala and the subsequent reduction of proinflammatory cytokine release (Lu et al., 2023). These mechanisms are associated with attenuated mechanical hyperalgesia in rodent models of DPN.

In addition, an experimental study investigated fluorocitrate and neurotropin as potential therapies for DPN via central astrocyte modulation (Liu et al., 2022). Fluorocitrate, a glial-specific metabolic inhibitor that disrupts Krebs cycle activity (Zhuang et al., 2025), and neurotropin, a biologic agent derived from vaccinia virus-inoculated rabbit skin (Sprumont et al., 1995), were evaluated in diabetic rats. Both agents reduced mechanical hypersensitivity and normalized astrocyte activation markers in the vlPAG when administered via intrathecal (fluorocitrate) or systemic (neurotropin) routes (Liu et al., 2022). Critically, these analgesic effects occurred without altering blood glucose levels, suggesting a glucose-independent mechanism of action centered on astrocyte regulation. These findings highlight astrocytes as potential therapeutic targets for DPN management.

Beyond astrocytic modulation, other compounds targeting neuroinflammation through different mechanisms have also shown efficacy. Thalidomide, a derivative of glutamic acid, exhibits immunomodulatory and anti-inflammatory effects (Millrine and Kishimoto, 2017). RVM microinjections of thalidomide in Zucker diabetic fatty (ZDF) rats significantly reduced mechanical allodynia and thermal hyperalgesia (Yang et al., 2016). The analgesic effects were correlated with localized suppression of pro-inflammatory mediators (TNF-*α*, IL-1β) and NF-κB signaling within the RVM microenvironment. However, systemic cytokine levels remained unchanged, indicating region-specific anti-inflammatory action rather than global immunomodulation. It should be noted, nevertheless, that despite these mechanistic insights, the clinical application of thalidomide is limited by its well-documented toxicity concerns (Matthews and McCoy, 2003).

In addition to direct anti-inflammatory approaches, receptor-based interventions have emerged as another promising strategy. Among these, glucagon-like peptide-1 receptor agonists (GLP-1RA), commonly used for type 2 diabetes, have shown additional potential in DPN (Dhanapalaratnam et al., 2024). In animal models, intracerebroventricular administration of GLP-1RA has been shown to alleviate thermal and mechanical allodynia in DPN rats and suppress microglial activation in the cortex and thalamus, suggesting that GLP-1RA attenuates DPN, likely through inhibition of NLRP3 inflammasome activation in brain microglia (Zhang et al., 2022).

Beyond neuroinflammation and receptor modulation, mitochondrial dysfunction has been increasingly recognized as a shared pathological mechanism in neuropathic pain, including DPN (Yu et al., 2025; Espinoza and Papadopoulos, 2025). In the ZDF rat model, chronic oral administration of NSI-189, a neurogenic compound, ameliorated indices of neuropathy by improving mitochondrial bioenergetics (Jolivalt et al., 2022). Specifically, NSI-189 enhanced expression of mitochondrial respiratory complex subunits (III and V) and restored the activities of complexes I and IV in the brain cortex, changes that were accompanied by improved memory function and synaptic plasticity. These findings suggest that mitochondrial protection may represent an additional therapeutic avenue for targeting CNS dysfunction in DPN, though clinical translation remains to be established.

In summary, brain-targeting compounds primarily act by modulating central neuroinflammatory pathways, engaging specific receptor targets, or protecting mitochondrial function. While preclinical findings are encouraging, further translational research is required to clarify their safety, efficacy, and clinical applicability in DPN.

### 5.3 Clinical translation and future perspectives

Collectively, the integration of neuroimaging and preclinical findings provides a mechanistic foundation that can inform clinical interventions in DPN. For example, evidence of cortical and subcortical reorganization has guided the application of non-invasive brain stimulation techniques such as rTMS (Zeng et al., 2020), while the identification of neuroinflammatory and mitochondrial pathways has stimulated the search for brain-targeting pharmacological agents (Kaur et al., 2025; Zhu et al., 2023). Although most compounds remain at the preclinical stage, these mechanistic insights highlight promising therapeutic avenues that may complement existing symptomatic treatments. Importantly, future clinical trials should be designed to bridge these translational gaps, incorporating neuroimaging biomarkers to stratify patients and monitor treatment responses (Zhu et al., 2023; Hermann et al., 2025). Such an approach may accelerate the development of mechanism-based and personalized therapies for painful DPN (Atmaca et al., 2024).

While the present review highlights CNS alterations in DPN, it is equally important to consider how patient heterogeneity shapes these changes and modulates treatment responses, which is critical for advancing personalized therapy (Yang et al., 2019; Sloan et al., 2021; Teh et al., 2021; Croosu et al., 2023; Bonhof et al., 2019). Variability in pain phenotypes (e.g., painful vs. painless DPN), diabetes duration, and comorbidities such as anxiety or depression can influence the progression of CNS alterations (Shillo et al., 2019; Rosenberger et al., 2020; Gore et al., 2005; Lee and Won, 2025). For instance, patients with longer disease duration or genetic predispositions may show more pronounced thalamic atrophy or altered somatosensory connectivity, leading to greater central sensitization (Teh et al., 2021). Such heterogeneity contributes to differential CNS plasticity and variable responsiveness to interventions: neuromodulation (e.g., rTMS) may benefit certain phenotypes, whereas pharmacological therapies may be less effective in those with comorbid depression or genetic variants affecting drug metabolism (Sloan et al., 2021; Zeng et al., 2020; Zhu et al., 2023; Bonhof et al., 2019). To address these challenges, future studies should prioritize patient stratification based on phenotypes, CNS biomarkers, and complementary measures such as quantitative sensory testing or neuroimaging profiles, thereby facilitating more tailored and effective therapies (Yang et al., 2019; Sloan et al., 2021; Zhu et al., 2023; Atmaca et al., 2024; Bonhof et al., 2019; Lee and Won, 2025).

## 6 Conclusion

DPN is not solely a peripheral disorder; it involves profound changes in the brain’s pain-processing and modulation networks. Structural alterations, such as cortical thinning and subcortical atrophy, along with functional disruptions in connectivity and excitatory–inhibitory imbalance, contribute to central sensitization and pain persistence. Neuroinflammatory processes driven by microglial and astrocytic activation further amplify neuronal dysfunction and exacerbate chronic pain states.

These diverse alterations converge within the pain matrix, integrating sensory, emotional, and cognitive aspects of pain. Maladaptive reorganization of this matrix in DPN provides a unifying explanation for how peripheral injury, central sensitization, and higher-order processes interact to sustain chronic pain.

Understanding the role of the pain matrix in DPN provides a foundation for developing targeted therapies. Interventions aimed at restoring functional connectivity, reducing neuroinflammation, and enhancing descending inhibition hold promise for addressing the brain mechanisms of pain. Future research should prioritize longitudinal studies to elucidate the progression of central changes in DPN and explore multimodal approaches that integrate peripheral and central treatments for optimal pain management.
